# T cell independent antibody responses with class switch and memory using peptides anchored on liposomes

**DOI:** 10.1038/s41541-024-00902-3

**Published:** 2024-06-22

**Authors:** Ásdís Hjálmsdóttir, Fabio Hasler, Ying Waeckerle-Men, Agathe Duda, María Pilar López-Deber, Maria Pihlgren, Marija Vukicevic, Thomas M. Kündig, Pål Johansen

**Affiliations:** 1 Department of Dermatology, University of Zurich, Zurich, Switzerland; 2 Department of Dermatology, University Hospital Zurich, Zurich, Switzerland; 3 AC Immune SA, EPFL Innovation Park EPFL, Lausanne, Switzerland

**Keywords:** Adjuvants, Drug development

## Abstract

Vaccines generally require T lymphocytes for B-cell activation and immunoglobulin class switching in response to peptide or protein antigens. In the absence of T cells, limited IgG class switch takes place, germinal centers are short-lived, and the B cells lack memory. Here, immunization of mice with liposomes containing 15mer peptides and monophosphoryl lipid A (MPLA) as adjuvant, induced T-cell independent (TI) IgG class switch within three days, as well as germinal center formation. The antibody responses were long-lived, strictly dependent on Toll-like receptor 4 (TLR4) signaling, partly dependent on Bruton’s tyrosine kinase (BTK) signal transmission, and independent of signaling through T-cell receptors, MHC class II and inflammasome. The antibody response showed characteristics of both TI type 1 and TI type 2. All IgG subclasses could be boosted months after primary immunization, and the biological function of the secreted antibodies was demonstrated in murine models of allergic anaphylaxis and of bacterial infection. Moreover, antibody responses after immunization with peptide- and MPLA-loaded liposomes could be triggered in neonatal mice and in mice receiving immune-suppressants. This study demonstrates T-cell independent endogenous B-cell memory and recall responses in vivo using a peptide antigen. The stimulation of these antibody responses required a correct and dense assembly and administration of peptide and adjuvant on the surface of liposomes. In the future, TI vaccines may prove beneficial in pathological conditions in which T-cell immunity is compromised through disease or medicines or when rapid, antibody-mediated immune protection is needed.

## Introduction

Activation of B cells and subsequent antibody production require CD4 T-helper (T_H_) cells when antigen is a protein. In contrast, bacterial polysaccharide and repetitive epitopes of viral particles can activate B cells by extensive cross-linking of antigen receptors without the help of T_H_ cells^1,2^. This results in rapid and intense antibody response against blood-borne and acute infections and is crucial for the early protection against pathogens, while IgG antibody responses to T-cell dependent (TD) antigens are slower^3^. TD and T-cell independent (TI) antibody responses start in lymph nodes with antigen binding to B-cell receptors (BCRs). TD antigens are internalized, processed and presented to cognate T cells on MHC class II molecules in secondary lymphoid organs, where activated T cells provide stimulatory signals to B cells, which differentiate into short-lived extra-follicular plasma cells, early memory cells, or germinal center (GC) B cells. In the GCs, IgM on B-cell surfaces undergo irreversible immunoglobulin class switch and affinity maturation, and the B cells differentiate into antibody-secreting plasma cells or long-lasting memory B cells^4–6^. In general, TI antigens do not induce GC formation or memory B cells^7^. Type-1 T (TI-1) antigens may activate B cells through signaling via Toll-like receptors (TLR) that recognize microbial ligands and induce a polyclonal, non-antigen-specific B-cell response^2^. Type-2 TI (TI-2) antigens are typically repetitive, e.g., bacterial capsular polysaccharides, which extensively crosslink BCRs and deliver persistent signals to the B cell through the cytoplasmic enzyme Bruton’s tyrosine kinase (BTK)^8^.

Immunization with the experimental TI-2 antigen NP-Ficoll can form GCs in transgenic mouse models with high numbers of high-affinity antigen-specific B cells^7,9^. In wild type (WT) and in T-cell deficient mice, NP-Ficoll induced short-lived GCs with limited affinity maturation and isotype class switch^7,9–11^. TI-associated GCs require extensive crosslinking of BCRs and are independent of CD40L or CD28 signaling. In TD-associated GCs, proliferating B cells (centroblasts) undergo somatic hypermutation in the dark zone of the GCs. Subsequently, the centroblasts exit the cell cycle as smaller, non-dividing and immunoglobulin-expressing centrocytes that migrate to the light zone, partly driven by expressed of CXCR5 that responding to CXCL13 produced by follicular dendritic cells (FDCs)^12,13^. In the light zone, the B cells encounter FDCs, follicular T_H_ (T_FH_) cells, and macrophages^6,14^ that results in selection and survival of high-affinity centrocytes and provides signals for differentiation of B cells to long-lived plasma and memory B cells^15,16^. In contrast, cells centrocytes that do not bind antigen on FDCs, T_FHs_ or macrophages will egress from the lymph nodes by sensing sphngosine-1-phosphate or succumb to apoptosis^17^. Of note, the dynamics of TD-associated GC cell migration may include a two-way system where some centrocytes can return to the dark zone for additional rounds of mutation and selection^18^.

A unique type of extra-follicular memory B cells has been identified in response to both TI-1 and TI-2 antigens^19–21^. However, recall responses to these TI antigens were only obtained with haptened polysaccharides and in murine models after adoptive transfer of already primed B-cells. However, the secondary activation of TI-memory B cells was typically down-regulated by antigen-specific antibodies, potentially as a self-tolerance mechanism to avoid antibody over-production. A further suggestion of TI-like B-cell memory was the observation that protection upon secondary infection could be provided by long-lived bone marrow plasma cells independent of T cells^22^.

The current study aimed to test the existence of TI-like B-cell memory after immunization with peptide and without using a murine transfer model. Indeed, immunization with short peptide antigens and a TLR4 ligand densely arranged on the surface of nano-sized liposomes activated B cells directly and independent of T cells. We observed the generation of long-lived and class-switched antibodies, B-cell memory and GC formation. The antibody responses showed characteristics of both TI-1 and TI-2 type reactions, being dependent on MyD88 and in part also on BTK signaling. This study demonstrates endogenous, T-cell independent and functional B-cell recall responses in vivo using peptide antigens.

## Results

### Peptide- and MPLA-loaded liposomes induced very fast antigen-specific sero-conversion with IgG class switch

**Fig. 1:**
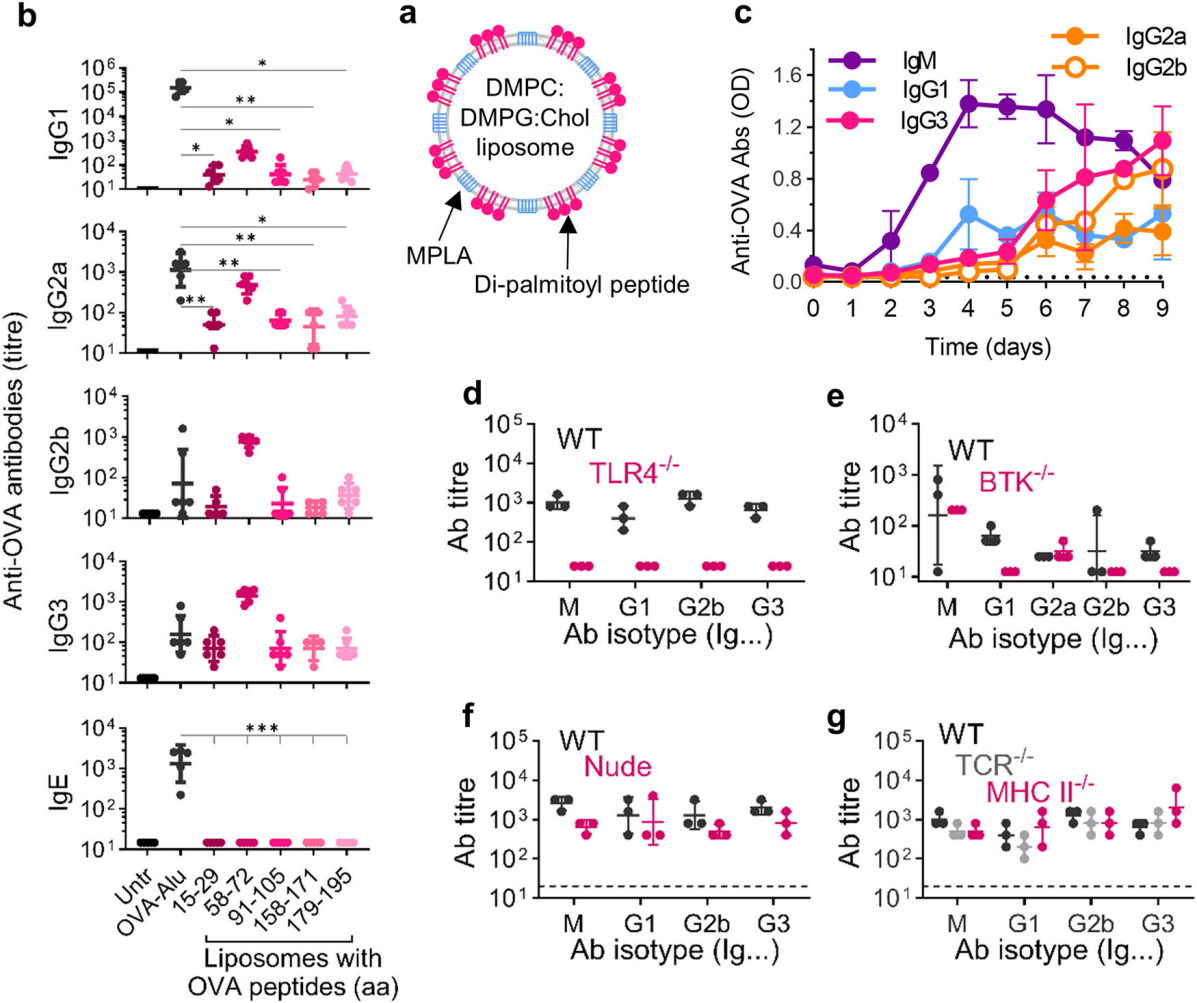
Peptide- and MPLA-loaded liposomes stimulate IgG class switch in vivo and independent on T cells, but dependent on TLR and BTK signaling. **a** Schematic representation of ovalbumin (OVA) peptide- and MPLA-loaded liposomes. The peptide is anchored in the liposomal membrane by palmitoyl chains coupled on both ends of the peptide. The adjuvant monophosphoryl lipid A (MPLA) is integrated in the liposomal membrane through six acyl chains. **b** OVA-specific antibodies determined in BALB/c mice (*n* = 6) six weeks after two immunizations (days 0 and 7) with the indicated OVA peptide-liposomes (OVAaa) or with OVA protein on aluminum (OVA-Alu). Data were analyzed using a Kruskal Wallis test comparing any group to OVA-Alum. **c** Rapid OVA-specific IgG class switch in BALB/c mice after injection single immunization with 10 µg OVA_58-72_ liposomes (*n* = 3). **d**–**g** OVA-specific antibody responses in **d** BALB/c wild-type (WT) and athymic (Nude) mice, **e** C57BL/6 WT, TCR-deficient (TCR^−/−^) and MHC class II-deficient (MHC II^−/−^) mice, **f** C57BL/6 WT and TLR4-deficient (TLR4^−/−^) mice or **g** CBA WT and syngeneic BTK-deficient mice (BTK^−/−^) six weeks after two immunizations with Lip-OVA_58-72_ as indicated above (*n* = 3-5). Antibody levels are expressed as reciprocal geometric mean (GM) ± GSD of titers **b**, **d**–**g** or as OD ± SD **c**. Data were representative of two to four independent experiments with comparable results. *p < 0.05; **p < 0.01; ***p < 0.001.

Immunization of WT BALB/c mice with peptide- and MPLA-loaded liposomes triggered OVA-specific IgG isotype switch with production of IgG1, IgG2a, IgG2b, and IgG3 subclasses (Fig. 1b). Among six tested preparations, the 15mer OVA_58-72_ was the most immunogenic; the antibodies were specific for the individual OVA-derived peptides loaded on the liposomes (Suppl. Fig. 1a). The levels of antigen-specific IgG2a and IgG2b antibodies secreted were comparable to the levels obtained using a TD vaccine based on OVA full protein and alum (OVA-Alu). OVA-Alu was more immunogenic for IgG1 production (*p* = 0.0022), while the OVA_58-72_-liposomes induced stronger IgG3 responses (*p* = 0.0043). While OVA-Alu stimulated OVA-specific IgE, the liposomal vaccines did not. Of note, immunization with a single injection of soluble OVA_58-72_ or OVA_179-195_ peptides admixed to MPLA-loaded liposomes did not induce detectable antibody responses (Suppl. Fig. 1b) demonstrating the importance of membrane-associated peptide for the stimulation of antibodies. However, mixing whole OVA to MPLA-containing liposomes produced fast and high antibody responses of all subclasses (Suppl. Fig. 1c). Due to its superiority, the liposomal formulation with OVA_58-72_ and MPLA (Lip-OVA_58-72_) was used in all subsequent experiments, and it stimulated IgM production within two days of immunization (Fig. 1c). The IgM response peaked on days 4–6. The IgG1 and IgG3 class switch was observed on day 3, and while serum IgG1 was stable thereafter, IgG3 kept rising through day 9. The IgG2a and IgG2b serum conversions were observed on days 5–6.

### Antibody responses displayed characteristics of type-1 and -2 T-cell independent responses

Lip-OVA_58-72_ triggered strong OVA-specific antibody responses in T-cell deficient athymic nude mice with immunoglobulin class switch to all IgG subclasses (Fig. 1d). The antibody responses were comparable to those determined in immune-competent BALB/c mice. T-cell independency was confirmed in MHC class II-deficient mice lacking mature CD4 T cells and TCR-deficient mice lacking both αβ and ɣδ T cells (Fig. 1e). Of note, OVA-Alu did not stimulate antibody responses in athymic nude mice (not shown).

We further investigated the nature of the second signal for T-cell independent B-cell activation. The adjuvant MPLA can bind TLR4, which is receptor for TI-1 antigens such as LPS. Indeed, antibody production was completely abrogated in TLR4-deficient mice immunized with Lip-OVA_58-72_ (Fig. 1f). In TI-2 antibody responses, a co-stimulatory signal for B-cell activation comes through the cytoplasmic enzyme BTK following crosslinking of the BCRs. We hypothesized that the high density of peptides on the liposomal cross-linked BCRs through BTK, and indeed, antibody production and class-switch was impaired in BTK-deficient mice immunized with Lip-OVA_58-72_ and only IgM and IgG2a antibodies were detected (Fig. 1g).

### Formation of germinal centers independent of T cells

**Fig. 2:**
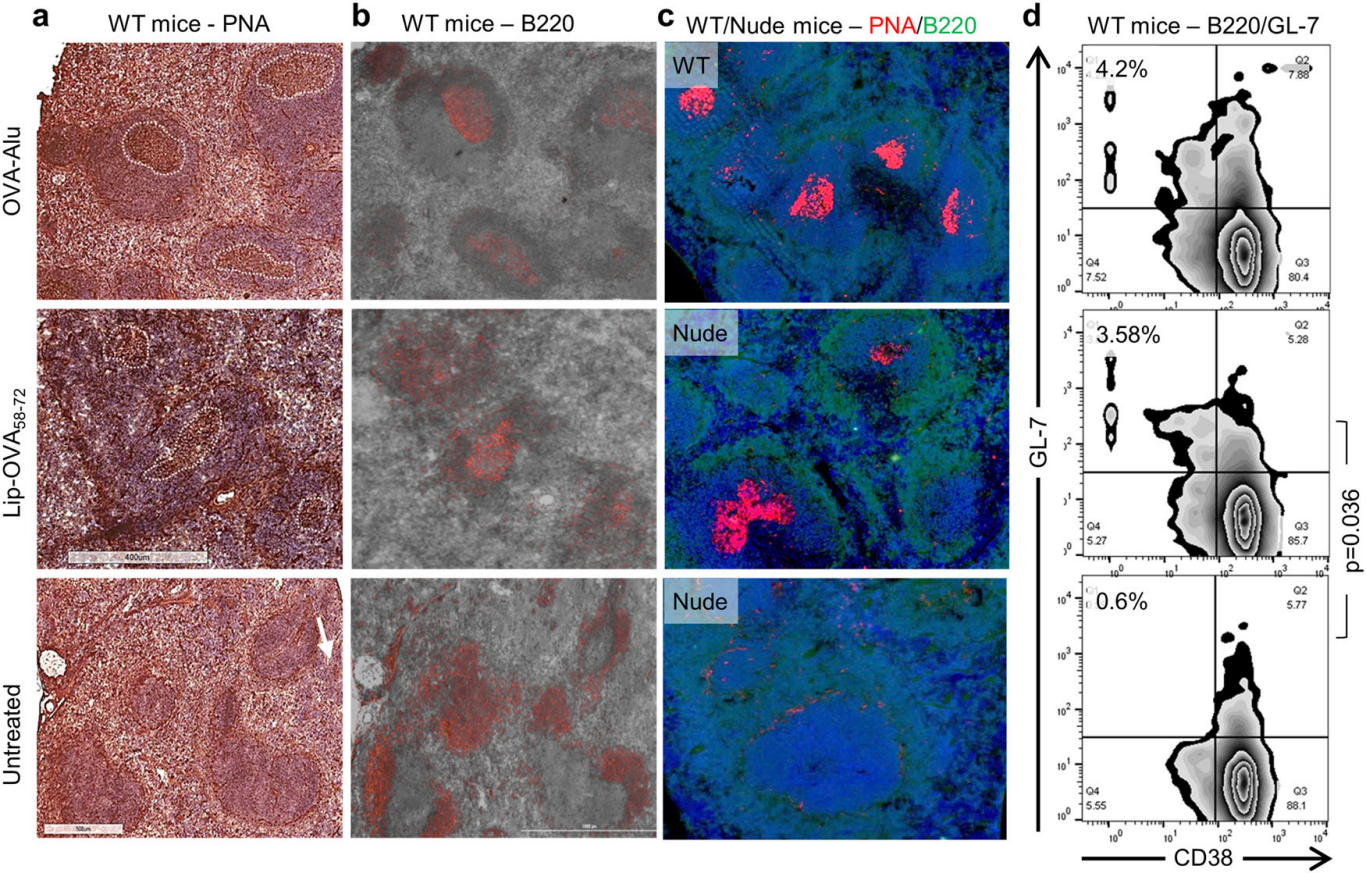
T-independent and long-lived germinal center formation. BALB/c WT and nude mice were immunized with 10 µg Lip-OVA_58-72_ or OVA-Alu. On day 5, mouse spleens from WT mice were analyzed histologically by staining with PNA **a** or for B220 **b**. Spleen from WT or nude were analyzed by immune fluorescence staining with PNA (red), B220 (green) and DAPI for determination of germinal center formation **c**. Splenocytes from immunized (*n* = 5) or untreated (*n* = 3) BALB/c mice were stained with B220, GL-7 and CD38 antibodies for flow cytometry, the germinal center being defined as B220-positive, GL-7-positive and CD38-negative as shown in zebra plots. The B-cell analysis was not antigen-specific. Lip-OVA_58-72_–treated (*n* = 5) and untreated mice (*n* = 3) were compared statistically using Mann-Whitney U test.

Immunization of BALB/c mice with Lip-OVA_58-72_ caused formation of GCs in the spleens follicles as determined from day five by histological staining of PNA (Fig. 2a) and B220 (Fig. 2b). Formation of GCs was also observed in athymic nude mice as early as three days after immunization and as determined by IF of PNA and B220 (Fig. 2c). Both in WT and nude mice, the GCs remained for at least 10 days (not shown). The GCs induced by peptide-loaded liposomes, in both BALB/c and nude mice, were of comparable in size, but generally lower in numbers when compared to GCs induced by immunization with a TD protein antigen (Fig. 2c). Flow cytometry analysis of spleens from BALB/c mice confirmed GC formation after immunization and revealed significantly increased expression of GL-7-positive CD38-negative B220-positive B cells compared to naive mice (Fig. 2d, *p* = 0.036, and Suppl. Fig. 2).

### T-cell independent B-cell memory and affinity maturation

**Fig. 3:**
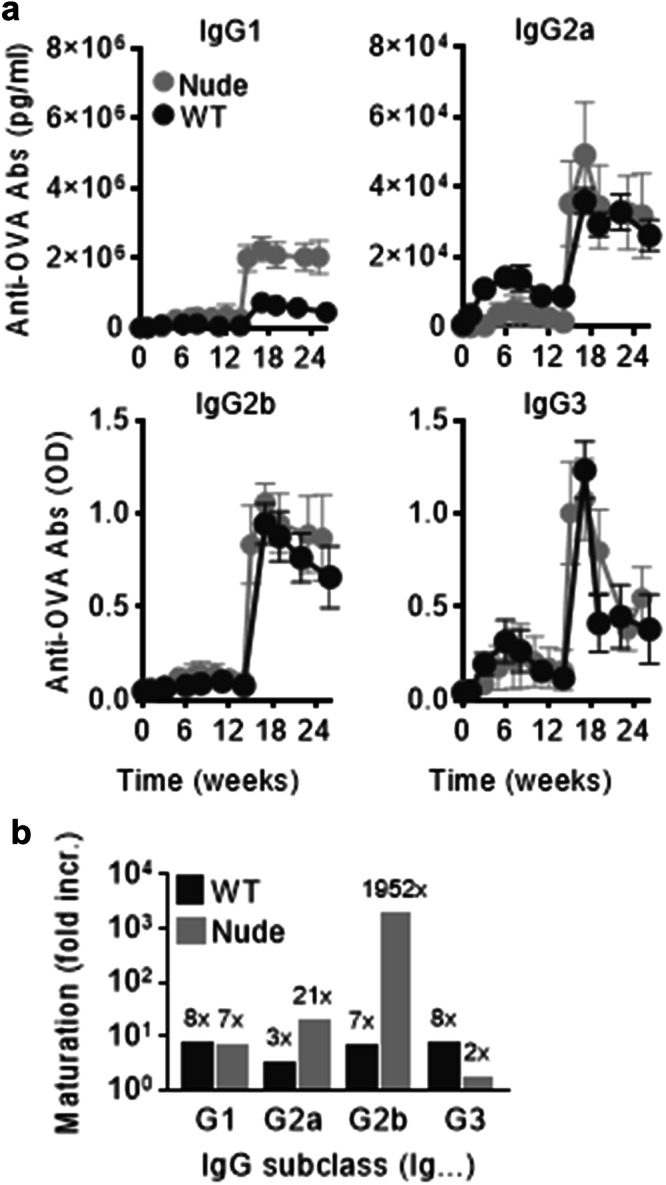
Antibodies are long-lived with affinity maturation and B-cell memory responses taking place independent on T cells. WT BALB/c and athymic nude mice were immunized with 10 µg Lip-OVA_58-72_ and boosted 14 weeks later. **a** Specific IgG1, IgG2a, IgG2b and IgG3 antibody responses to the whole OVA were measured at the indicated time points and the results are expressed as mean ± SD (*n* = 5). **b** Avidity of the measured antibodies on week 23 after immunization as analyzed by competitive ELISA. The number above the bars indicate the avidity fold increase between weeks 1 and 23.

After a single injection of the peptide- and MPLA loaded liposomes, high levels of OVA-specific antibodies of all IgG subclasses were detected in serum for at least 14 weeks, and the antibody longevity was not dependent on T cells (Fig. 3a). A strong and T-cell independent boost of all IgG subclasses was observed upon secondary immunization. The results revealed that neither antibody levels nor memory properties were compromised by the lack of T cells as observed in athymic nude mice, and no statistical difference was observed between athymic nude and athymic mice.

The avidity of antibodies from immunized BALB/c and nude mice was evaluated by competitive ELISA as a measure of affinity maturation. The tested sera were collected one week after the primary immunization and eight weeks after the secondary immunization (week 23). The antibody avidity increased from week 1 to week 23 for all antibody subclasses and all treatment groups (Fig. 3b). The primary avidity measured after immunization with OVA on alum was typically slightly higher than with peptide-loaded liposomes (not shown), but the antibody avidity, especially IgG2a and IgG2b, increased strongly in athymic nude mice following immunization with OVA_58-72_ and MPLA liposomes.

### Liposome triggered cytokine and IgM secretion from B cells in vitro

**Fig. 4:**
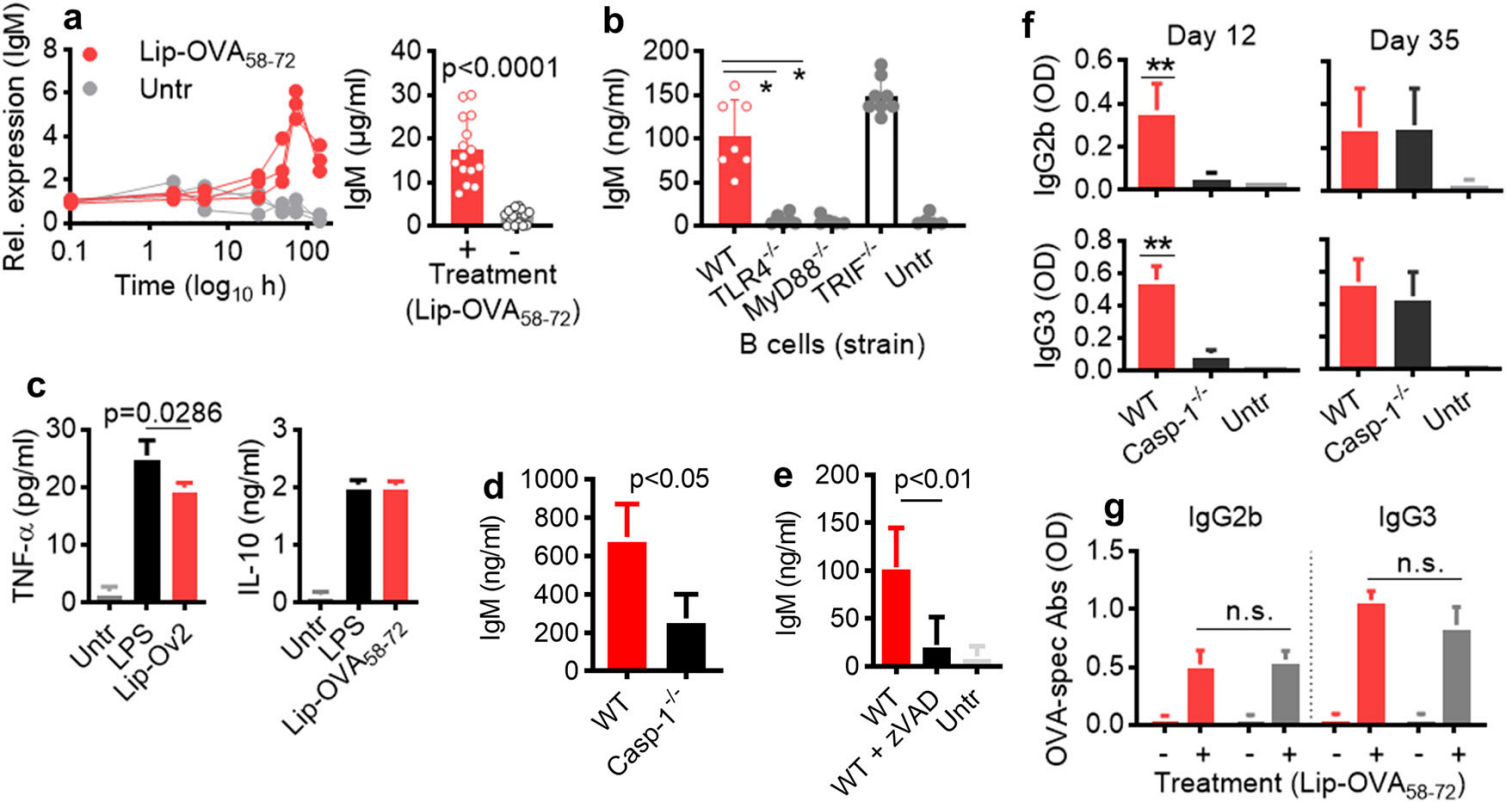
IgM and cytokine secretion from B cells are dependent on inflammasome activation. **a**–**f** Naive B cells from spleens mice were stimulated in vitro with 10 µg/ml Lip-OVA_58-72_ for two days. **a** Cells and supernatants were harvested to measure the IgM expression and total IgM by PCR and ELISA, respectively. Untreated B cells were used as negative controls. **b** IgM secretion of B cells of C57BL/6, TRL4-, MyD88- or TRIF-deficient mice after in vitro stimulation with Lip-OVA_58-72_. **c** TNF-α and IL-10 secretion of B cells of C57BL/6 mice after stimulation with Lip-OVA_58-72_ or LPS (5 µg/ml) as compared to untreated cells (*n* = 5). **d**, **e** IgM secretion from Lip-OVA_58-72_-stimulated B cells of WT or caspase 1-deficient mice **d**, or from Lip-OVA_58-72_-stimulated WT B cells in the presence or absence of pan caspase-inhibitor z-VAD **e**. **f** OVA-specific IgG2b and IgG3 responses (OD_1:150_) in WT C57BL/6 or caspase 1-deficient mice (*n* = 5) as measured on day 12 (top panel) and day 35 (bottom panel) after immunization with Lip-OVA_58-72_. **g** OVA- (gray bars) specific IgG2b and IgG3 responses (OD_1:150_) in WT or ASC-deficient (ASC) mice (*n* = 5) after immunized with Lip-OVA_58-72_. Means and SD are shown. Kruskal Wallis test or Mann-Whitney U test were applied for statistical analysis. *p < 0.05; **p < 0.01; n.s.: not significant.

Purified B cells from spleens and lymph nodes of naive mice produced IgM transcripts and secreted total IgM in vitro within two days of stimulation with Lip-OVA_58-72_ (Fig. 4a and Suppl. Fig. 3). No secretion of IgM was observed in B cells from TLR4- or MyD88-deficient mice, while TRIF-deficient mice were IgM competent (Fig. 4b), further underlying TI-1 immune stimulation by the liposomal preparations. Moreover, Lip-OVA_58-72_ stimulated secretion of inflammatory cytokines such as TNF-α and IL-10 (Fig. 4c). The IgM and cytokine secretion was not dependent on peptide since liposomal preparations with MPLA but without peptide stimulated comparable amounts of IgM and cytokines (data not shown). Collectively, the results suggest that the MPLA-decorated liposomes also triggered inflammatory responses in B cells in the absence of innate cells, and that the B cells may possibly use this inflammatory bystander response in an autocrine fashion for antibody production and isotype switching.

### Antibody responses were partly dependent on inflammasome activation in vitro and in vivo

The activation of TLRs has been associated with activation of inflammasome and subsequent caspase-1 activation in various immune cells. Caspase-1 mediate the secretion of inflammatory cytokines such as IL-1β. Also, adjuvant effects have been explained through inflammasome activation. We therefore tested the response to liposomal peptide vaccines in purified caspase-1-deficient B cells in vitro. The IgM secretion was strongly reduced in Lip-OVA_58-72_-stimulated B cells from caspase-1 deficient mice as compared to WT cells (Fig. 4d). The same was observed when WT B cells were stimulated with Lip-OVA_58-72_ in the presence of the pan caspase inhibitor z-VAD (Fig. 4e). Also in vivo, the stimulation of antigen-specific IgG responses were initially impaired in caspase 1-deficient mice as compared to WT mice (Fig. 4f). However, at later time points, no difference in the serum level of antigen-specific antibodies was observed. Also in mice deficient for ASC, the caspase-1 recruiting adaptor protein in all inflammasome, isotype switch to all IgG subclasses was observed after immunization with Lip-OVA_58-72_, and the long-term antibody levels was not impaired by the lack of ASC (Fig. 4g).

### T-cell independent vaccines as therapeutics by immune suppression

**Fig. 5:**
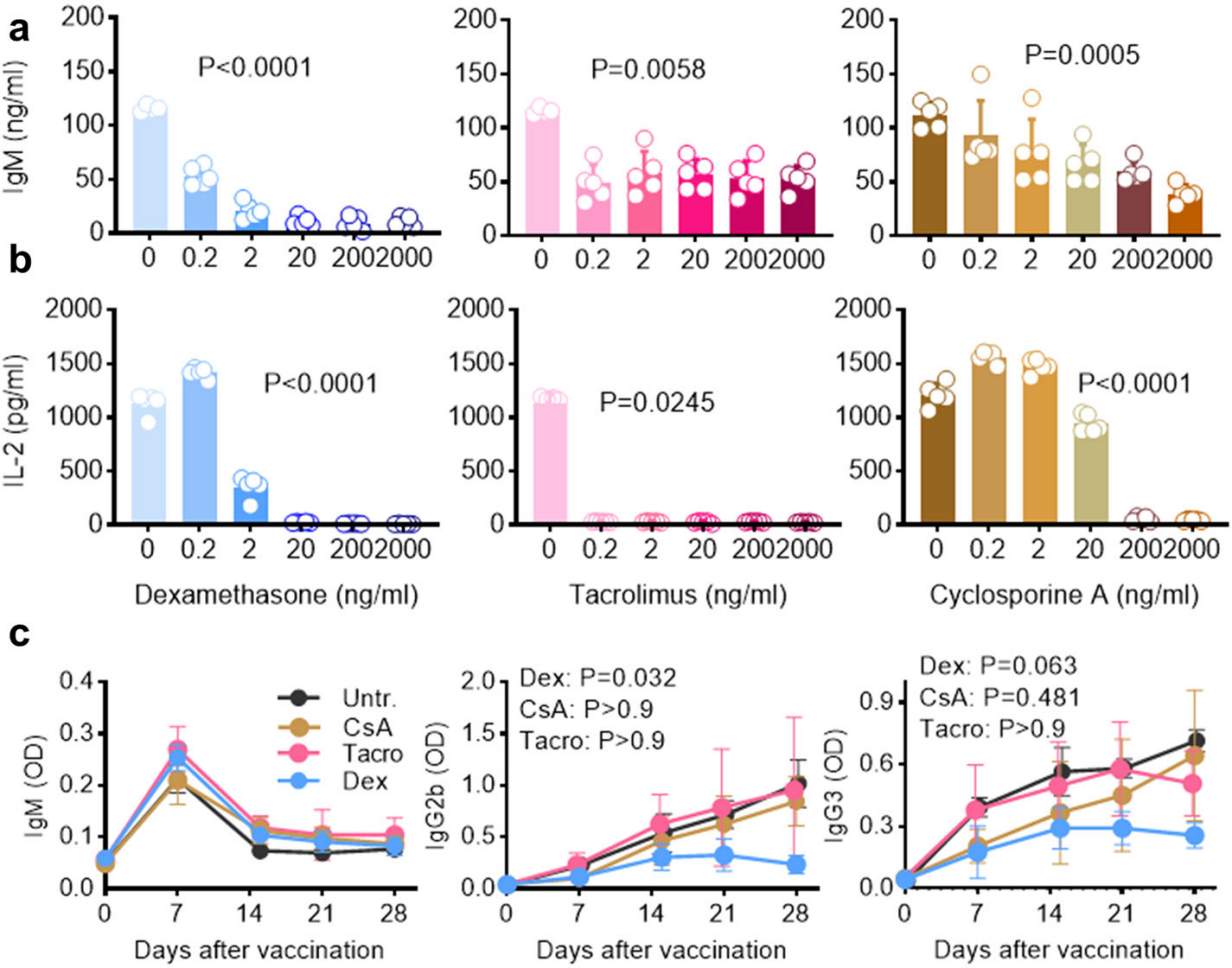
Overcoming immune suppression with T-cell independent vaccines. Cultures of purified B cells **a** or whole splenocytes **b** from C57BL/6 mice were stimulated two days in vitro with Lip-OVA_58-72_ **a** or concanavaline A **b** in the presence of immunosuppressive dexamethasone (blue), tacrolimus (pink) or cyclosporine A (ochre). IgM from B cells **a** and IL-2 from T cells **b** secretion were measured in the culture supernatants by ELISA. The indicated p values were calculated by Kruskal Wallis and testing if treatment affects readout. **c** OVA-specific IgG2b and IgG3 responses in C57BL/6 mice immunized with Lip-OVA_58-72_ in the absence or presence of indicated immunosuppressive drugs (*n* = 4–6). Means and SD are shown. The area under the curve for each group was calculated and compared to untreated (Untr) mice by Kruskal Wallis with Dunn’s multiple comparison tests.

One field of application for vaccines with TI antigens may be in vaccination or immunotherapy of immunocompromised or immune-suppressed patients. Since many immune suppressive agents are active at the level of T cells, we hypothesized that peptide- and MPLA-loaded liposomes could break sensitivity to several immune suppressive drugs. To this end, mouse B cells purified from spleens (Suppl. Figure 3) were stimulated with liposomal preparation in vitro in the presence of the immune-suppressive drugs dexamethasone, tacrolimus or cyclosporine A. When B cells were stimulated with liposomes containing OVA peptides, peptide-specific IgM production was abrogated using dexamethasone (Fig. 5a). The immune-suppressive effect of the drug was also confirmed by measuring IL-2 secretion from whole mouse splenocytes upon stimulation with the mitogen concanavaline A (Fig. 5b). Both for B and T cells, dexamethasone suppressed immune reactivity at a dose of 2–20 ng/ml. While dexamethasone binds the glucocorticoid receptor on all immune cells and thereby mediates changes in gene expression that lead to multiple downstream immune-suppressive effects, tacrolimus and especially cyclosporine A specifically inhibits T-lymphocyte activation. Tacrolimus completely abolished IL-2 secretion in T cells at 0.2 ng/ml, whereas cyclosporine A exerted this effect at 200 pg/ml (Fig. 5b). In contrast, the IgM production from B cells was only reduced by 2–4 fold at the same drug concentrations (Fig. 5a). In addition, the TNF-α secretion of B cells after Lip-OVA_58-72_ stimulation was strongly suppressed with dexamethasone, but not with cyclosporine A (data not shown). To test if the vaccines also resisted immune suppression in vivo, mice were immunized with Lip-OVA_58-72_ in the absence and presence of any of the immune-suppressive drugs. The antibody production was significantly reduced by dexamethasone treatment, but not by treatment with tacrolimus or cyclosporine A (Fig. 5c).

### Potential neonatal use of TI vaccines

**Fig. 6:**
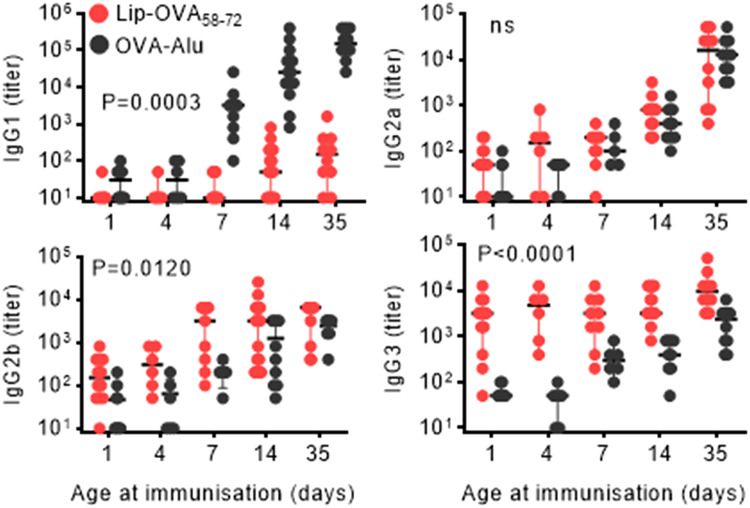
Neonatal immunization with TI liposomal OVA_58-72_ or TD aluminum OVA vaccines. BALB/c pups (*n* = 5-12) from naive mothers were immunized with either Lip-OVA_58-72_ (red) or OVA-Alu (black) at 1, 4, 7, 14 or 35 days of age. OVA-specific IgG subclass responses were determined two weeks later. Data are presented as geometric mean with 95% Cl. Mixed-effect model with Geisser-Greenhouse correction was used to compare statistically antibody responses between the two groups. ns: not significant.

Immunization or vaccination of neonatal children is often contraindicated due to a not-yet matured immune system^23^. We therefore tested whether liposomal TI vaccines could be applied in newborn mice for stimulation of antigen-specific antibody responses and if yes, at what age this was feasible. For this, we performed a single immunization and no boost. The secondary aim was to compare the response with the best possible and relevant children vaccine, which would be an alum-based vaccine. Mice were immunized at age 1, 4, 7, 14, or 35 days and antibodies in serum were measured two weeks post injection: of note, mice were kept with their mothers and siblings until age 21 days (Fig. 6). A clear IgG1 sero-conversion could be stimulated in 7 days or older mice for the TD vaccine OVA-Alu and in 14 days or older mice for the liposomal TI vaccine. For IgG2a, sero-conversion was obtained in mice 7 days or older for both TD and TI vaccines. After immunization with the TI vaccine, IgG2a sero-conversion was observed in 4 out of 6 mice (67%) of age 4 days and in 9 out of 12 mice (75%) of age 1 day. Three out of 10 mice (30%) of age 1 day showed IgG2a antibodies upon TD immunization. IgG2b sero-conversion was observed in 11 out of 12 mice (92%) of age 1 day and in 6 out of 6 mice (100%) of age 4 days immunized with the liposomal TI vaccine, while the corresponding frequencies for the TD vaccine OVA-Alu were 4 out of 10 (40%) and 3 out of 6 (50%). Finally, all one-day old mice produced antigen-specific IgG3 responses to the TI peptide vaccine, and the response titer was approx. 100 fold higher than in mice immunized with the TD protein vaccine.

### Antibodies induced with peptide- and MPLA-loaded liposomes have protective functions in mouse models or allergic anaphylaxis and bacterial infection

**Fig. 7:**
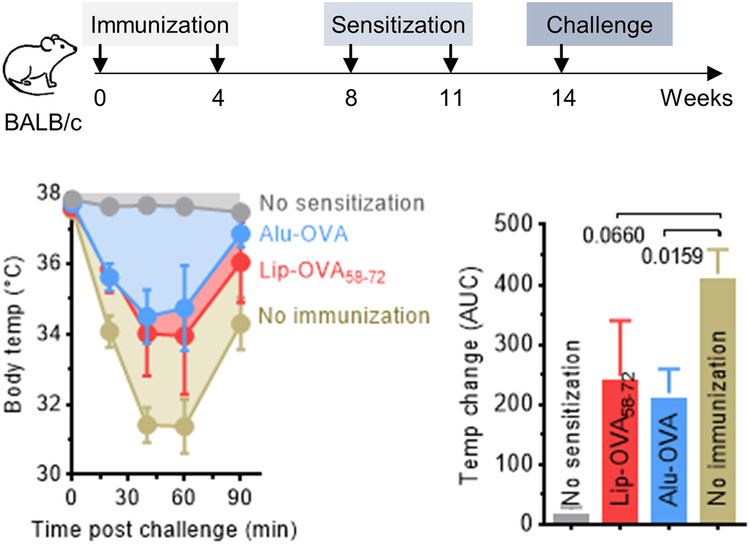
Prophylactic immunization with Lip-OVA_58-72_ reduced anaphylactic responses in OVA-allergic mice. BALB/c mice (*n* = 5) were immunized twice (weeks 0 and 4) with Lip-OVA_58-72_ or OVA protein on alum (Alu-OVA) or left untreated (No immunization). On weeks 8 and 11, all mice were subject to sensitization with OVA protein on alum. One group of non-immunized mice were left non-sensitized (No sensitization). On week 14, all mice were challenged intraperitoneally with soluble OVA and the body temperature monitored as a measure for allergic anaphylaxis; the integrated AUC for the body-temperature curves was calculated using pre-challenge temperature as baseline. Results are expressed as mean + SEM and analyzed using Kruskal-Wallis tests comparing the two immunized groups to the non-immunized group. Statistical *p* values are indicated.

The biological function of antibodies induced by peptide-loaded liposomes was further tested in a murine model of allergic anaphylaxis^24^. Briefly, mice were immunized s.c. with liposomes containing OVA_58-72_ and MPLA, then IgE-sensitized to OVA by i.p injection of OVA and alum, and finally challenged with a high dose of aqueous OVA (Fig. 7). Sensitized mice typically react with an anaphylactic shock manifesting as a drop in body temperature due to IgE-mediated and histamine- and mast-cell-dependent arterial hypotension and vasodilation^25^ and effects on thermoregulatory neural circuit^26^. However, mice that received prophylactic immunization with Lip-OVA_58-72_ prior to sensitization reacted with less anaphylactic symptoms and less severe temperature drops than did non-immunized mice. To rule out any role of T cells in the observed protection against anaphylaxis, serum from OVA-sensitized BALB/c mice was also transferred to immunized athymic nude mice twenty hours before the OVA challenge (Suppl. Fig. 4). The average temperature drop following challenge was lower in immunized than in non-immunized mice.

**Fig. 8:**
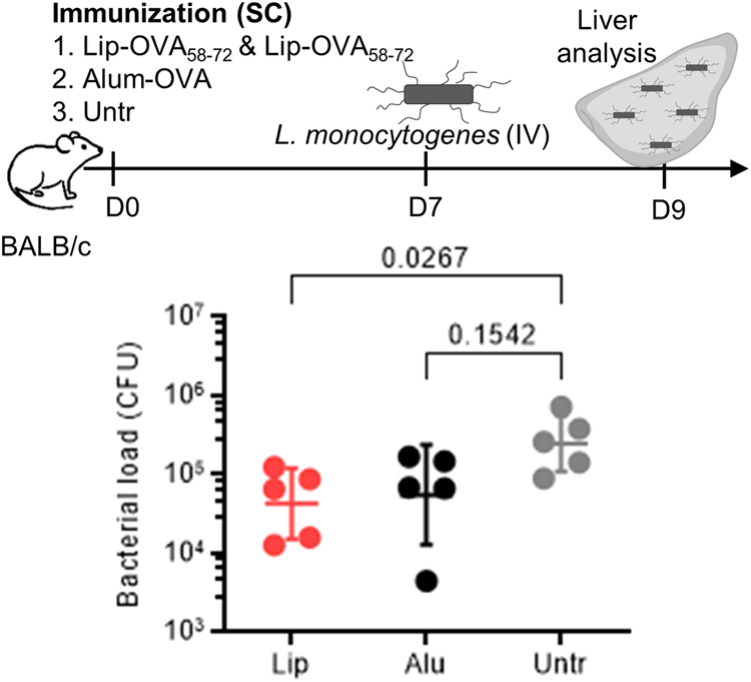
Immunization with liposomal TI vaccines reduced bacterial infection in mice. BALB/c mice (*n* = 5) were immunized with a combination of Lip-OVA_58-72_ and Lip-OVA_179-195_ (Lip), with OVA protein on alum (Alu) or left untreated. On day 7, all mice were infected with OVA-expressing *L monocytogenes*. On day 9, the mice were euthanized and the bacterial burden in liver was determined by plating tissue homogenates on agar and calculating CFU. Geometric means are shown and the results were analyzed using Kruskal Wallis tests comparing the two immunized groups to the non-immunized group. Statistical *p* values are indicated.

Due to the fast onset of antibodies after immunization with TI antigens, one may anticipate the use of such vaccines in infectious outbreaks of endemic or pandemic nature. We therefore tested the biological function of immunization-induced antibodies in an infectious model using OVA-expressing *L. monocytogenes*. As shown in Fig. 8, Lip-OVA_58-72_-immunized mice displayed significantly diminished bacterial loads in liver within one week of the immunization. The anti-bacterial effect was non-inferior to that of immunization with OVA and alum. Also a long-term function of TI antibodies was could be demonstrated, as mice infected six months post-immunization had lower bacterial burden compared to non-immunized mice (Suppl. Fig. 5a) while still having high levels of antigen-specific antibodies (Suppl. Fig. 5b).

## Discussion

The current study demonstrates that immunization with liposomes loaded with peptide antigens and the TLR4 ligand MPLA stimulated B cells fully independent on T cells. Moreover, the long-lasting IgG responses could be boosted months after the primary immunization. The study describes in vivo recall memory B-cell responses after immunization with a synthetic TI peptide vaccine. The switch to all IgG subclasses took place within four days of immunization in athymic nude mice and in mice deficient of αβ and ɣδ T cells or of MHC class II. The antibody responses displayed characteristics of both TI-1 and TI-2, since depending both on TLR4 signaling through MyD88 and on BTK signal transmission. GCs formed three days after immunization and were still visible after three weeks, and IgG affinity maturation was evidenced by the increased avidity over time. The TI antibodies were found to be functional using murine models of allergy and infection.

TI antigens do not require CD4 T_H_ cells for elicitation of antibody responses. Some TI-1 antigens, such as LPS, can stimulate B cells to proliferate in vitro through TLR signaling, resulting in antigen non-specific antibodies. However, efficient stimulation of antigen-specific antibody responses to TI-1 antigens require concomitant stimulation of BCRs and TLRs, both in vitro and in vivo^7^. In contrast, TI-2 antigens elicit antigen-specific antibody responses through extensive cross-linking of 10–20 BCRs^27–29^. By these means, repetitive polysaccharide antigens on gram-positive bacteria can elicit potent IgM responses, but limited class switch in the absence of T_H_ cells^20,21^. Viral envelopes also have highly organized protein structures and can elicit TI IgM and IgG antibodies, especially IgG3. However, antibody responses to non-replicating protein antigens, as in subunit vaccines, are characterized by IgM antibodies with no class switch to IgG, IgA or IgE in the absence of T_H_ cells^30–32^.

TD murine immune responses usually involve all IgG subclasses, but their relative abundance varies as a result of the prevailing cytokine environment, which again is affected by interactions of many cell populations within the lymphoid tissues^33^. We found that peptide- and MPLA-loaded liposomes stimulated predominantly IgG2 and IgG3, while the TD antigen mostly elicited IgG1 production. Also using liposomes loaded with MPLA and a 15mer amyloid-β peptide low levels of IgG1 and high levels of IgG3 were observed^34^. MPLA is a detoxified derivative of lipid A and LPS, a TLR4 ligand, and it is approved for use in human vaccines; other approved adjuvants are salts of aluminum salts, QS21, MF59 and CpG, alone or in combinations^35^. Simultaneous activation of TLRs and BCR further strengthens the B-cell activation and result in SYK-mediated activation of the NF-κB pathways^30,36–38^. Our study demonstrates that a high density of peptides and MPLA on liposomes provides strong enough signals to trigger directly immunoglobulin class switching in B cells. The broad distribution of resulting IgG subclasses is atypical for TI antibody responses, indicating a thus far unknown mechanism.

The antibody response induced by immunization with peptide-loaded liposomes displayed characteristics of both TI-1 and TI-2 responses. TI-1 antigens require TLR signaling to induce non-specific and polyclonal antibody responses^2^, while TI-2 antigens may use TLR as a second signal for induction of antigen-specific antibody responses^1^. In the current study, this second signal is most likely provided by MPLA, because the TI antibody response required that peptide and MPLA be incorporated into the surface of the same individual liposomes, suggesting that peptide and MPLA bind the same B cell and that antigen-presenting cells are not involved. MPLA can bind to either TLR4 or CD14, both known LPS receptors. Here, antibody production was abrogated in TLR4-deficient mice, and we have shown that antibody responses and IgG class switch was independent of CD14 co-stimulation^34^. Moreover, the antibody responses were unaffected in TRIF-, but abolished in MyD88-deficient mice, as observed for the two peptides (OVA_58-72_ and OVA_179-195_) tested in MyD88- and TRIF-deficient mice. In contrast, liposomes loaded with the amyloid-β 1–15 peptide stimulated antibody responses in a TRIF-dependent and MyD88-independent manner^34^. It is currently unclear what dictates whether TLR4 utilizes the MyD88 or TRIF signaling pathway. Most studies on TLR4 signaling has been performed on DCs and macrophages (see reviews^39,40^). In B cells, LPS and its immunogenic moiety lipid A have been found to stimulate TLR4 signaling through MyD88^41–43^, while in the presence of IL-4, TRIF signaling can become more important^41,44,45^. In contrast to TI-1 antigens, TI-2 antigens require intracellular signaling through BTK, following crosslinking of BCRs^2^. With the peptide-loaded liposomes, IgM antibody production as well as IgG2a class switch were surprisingly normal in BTK-deficient mice, while no class switch to IgG1, IgG2b, and IgG3 was observed. This result may suggest that BTK may play distinct roles in class switch to different subclasses. As for now, we do not know the mechanism behind the induction of antibody responses in BTK-deficient mice. However, while human B cells do not progress beyond the bone marrow in BTK-deficient persons, BTK is not required for B-cell maturation in the bone marrow in mice, and mouse B cells transport to the periphery, although at lower numbers than in wild type mice^46^. Moreover, TI-responses to infections are typically mediated by innate-like B1 cells, and BTK-KO mice have been shown unable to respond to TI-antigens due to lack B1 B cells and natural IgM^47^. However, in conditional BTK-KO mice, antibody response was demonstrated^48^. Hence, the lack of BTK may impair function of B cells during development of B1 cells. It was concluded that B-cell signaling is differentially regulated by BTK for development, survival and function, raising the possibility that BTK may also be dispensable for the function of B1 cells^48^. Although we are currently not able to pinpoint the exact mechanism on how a dense array of beta-sheet peptides on MPLA-containing liposomes trigger antibody responses independent on BTK, the observation of such immune responses may incentive further investigations using the same or similar models as to understand better the possibilities and limitations of BTK-independent B-cell stimulation.

Although, TI antigens are not expected to cause GC formation, somatic hypermutation, or B-cell memory, immunization with peptide-loaded liposomes enabled T-cell independent GC formation and increased IgG avidity. A few studies have reported short-lived and small GCs in the absence of T cells, but these studies used nitrophenyl conjugated to polysaccharide (NP-Ficoll) as antigen in TCRβδ^−/−^ mice^10^ and transgenic mice with high numbers of NP-specific B cells^7,9^. In the current study, we demonstrate that the GCs were T-cell independent and had a long lifespan after a single immunization and that TI-associated GCs were marginally smaller and less frequent than GCs formed in response to control immunization with TD antigens. We also showed that the GC formation was independent on CD28 and CD40 ligation^34^.

In TD-associated GCs, T cells are not needed until the stage of centrocyte selection in the light zone, after which a proportion of positively selected cells cycles back into the dark zone, as centroblasts, for further somatic hypermutation. The centroblasts likely undergo a finite number of divisions and then run out unless the pool is renewed^49^. This could explain the sudden termination of the NP-Ficoll-induced TI GC reaction as it collapses by massive B-cell apoptosis a few hours after the dark zone and light zone form, due to lack of positive selection and renewal of the centrocyte pool in the absence of T_FH_ cells^9^. Interestingly, we found peptide-induced GCs were still visible on day seven in TCR-deficient mice^34^, as long as 20 days in WT mice, and 10 days in athymic mice. Although more work is needed to find out why GCs survive in the absence of positive selection by antigen-specific T cells, NF-κB has been shown to control GC maintenance and differentiation in TD responses^50^ through MyD88-dependent TLR4 signaling^51^. GCs induced by non-peptide TI antigens typically have no or only limited mutations^9,52^. In contrast, we found increased avidity of each individual IgG subclass after immunization with peptide-loaded liposomes, suggesting hypermutation. Interestingly, a booster immunization with the alum-adsorbed and T-cell dependent protein vaccines increased the antibody avidity further, indicating that affinity maturation is completed earlier for the TI antigen. The affinity maturation of B cells can take place both in GCs and, in the absence T cells, in the extra-follicular space^11,53,54^. In fact, T-cell independent expression of AID, an enzyme involved in somatic hypermutation, was observed during B-cell development in a process involving both BCRs and TLRs^55^. This result supports a mechanism of action by which peptide- and MPLA-loaded liposomes, which also induces expression of AID^34^, may enable somatic hypermutation and affinity maturation independent of CD4 T_H_ cells.

Antibody responses to the peptide- and MPLA-loaded liposomes had a fast onset, while IgG class switch after immunization with TD antigens starts much later^56,57^. Moreover, IgG levels remained high for months after immunization and secondary immunizations demonstrated a memory recall response as IgG levels were boosted beyond the levels determined after the primary immunization. To our knowledge, this has never been described for TI peptide antigens. Memory B cells have been shown to be inducible with haptenated TI antigens, however, recall antibody responses could only be observed when primed B cells were adoptively transferred to naive, T-cell deficient recipients, before secondary antigen exposure^20,21,58–61^. Direct memory response has not been shown in the animal it was originally generated in^19^, and the TI B-cell memory has been found to be regulated through negative feedback by antigen-specific antibodies^21,59^. A marginal increase in IgG antibody-forming cells was reported upon secondary immunization with NP coupled to αCD180 antibody in CD40 deficient mice^62^. However, this IgG antibody response was shown to be partially dependent on T cells, as the titers were impaired in CD40- and TCR-deficient mice and hardly detectable in MHC II-deficient mice.

With TD antigens, an inflammatory environment is typically produced by concerted activity of various cells involved in shaping the overall immune response. Such cells include myeloid-derived antigen presenting cells such as macrophages and DCs, but also stromal cells, endothelial cells and epithelial cells. In response to antigen, adjuvants as well as danger (DAMPs) or pathogen (PAMPs) associated molecular patterns, these cells produce various cytokines that are supportive of the following innate and adaptive immune responses. With TI antigen, B cells have to bypass these supportive reactions or to take over many of the functions of the other cells. Indeed, we observed that purified and naive B cells produce high levels of the prototype pro-inflammatory cytokine TNF-α upon stimulation with liposomal in vitro. In response to vaccine, TNF-α is typically produced by APCs and other cells by signaling through the pan-inflammatory transcription factor NF-kB^63^, and the cytokine is central in activating effector functions or migratory functions in tissues cells or in draining lymph nodes. Through MPLA, we expect the TNF-α production in B cells in vitro to be associated with TLR signaling that is known to be an integral part of B-cell receptor signaling^64^. Activation of TLR have also been shown to trigger IL-10 production in B cells^65^, and we also find that the liposomal vaccine triggered strong production of IL-10 in murine B cells in vitro. Hence, the liposomal-based TI vaccine may in part exert their function by allowing the production of these and other cytokines by B cells, hence, bypassing the function normally required by other cells.

Interestingly, Lip-OVA_58-72_ stimulation of antibody production in vitro was strongly dependent on inflammasome activation, as culturing of B cells with Lip-OVA58-72 in the presence of the pan inflammasome inhibitor zVAD abrogated antibody production. It has also been shown that the inflammasome NLRP3 are activated in B cells by engagement of B-cell activating factor (BAFF) to its BAFF receptor^66^. BAFF is member of a TNF family and its signaling to NLRP3 involves NF-κB as well as caspase-1 activation. These findings revealed an important role of inflammasome activation in B-cell function and especially the associated homeostasis. In line with this, we found that antibody production was impaired in caspase-1-deficient B cells stimulated in vitro with Lip-OVA_58-72_. It has been suggested that certain TI antigens can trigger B-cell responses dependent on inflammasome activation. This canonical stimulation of the NLRP3 inflammasome by fungal β-glucan antigens triggered IgM production in vitro^67^. Equivalent to the second signal most likely triggered by MPLA stimulation of TLR4 in our study, the latter study applied CpG for stimulation of TLR9. However, when caspase-1- or ASC-deficient mice were immunized with Lip-OVA_58-72_, IgG class switching was not impaired and the overall antibody production only initially affected, suggesting that inflammasome activation is dispensable in TI-antibody production in vivo after stimulation of B cells with ip-OVA_58-72_.

The lack of B-cell recall responses in the absence of T cells raises the question how B cells and antibodies can be protective upon secondary infection if they are so tightly controlled. In responses to TD antigens, a second memory compartment of long-lived bone-marrow plasma cells is generated in addition to memory B cells^68,69^. The bone marrow plasma cells have been believed to be strictly T-cell dependent and require formation of GCs^70^. Yet, the TI-2 antigen *S. pneumoniae* capsular polysaccharide generated long-lived bone marrow plasma cells secreting protective IgM and IgG^22^. Nonetheless, the plasma cells generated by TD and TI antigens are functionally different, suggesting that the memory plasma cell compartment is heterogeneous^22^. It is unclear when the memory compartment comes into play upon secondary exposure to antigen, and the contribution of TI memory B cells and TI memory plasma cells may vary depending on antigen type and persistence as well as the route of antigen entry.

Vaccination is the most effective measure to prevent against infections. Protective immune responses often correlate with the affinity of neutralizing antigen-specific antibodies, for which reason the key to success is the generation of memory B cells. In certain situations, it would however be important to stimulate B-cell responses without involvement of T cells. Patients suffering from T-cell deficiencies, such as HIV, SCID, DiGeorge or Wiskott-Aldrich syndromes, or the elderly could benefit from TI vaccines^71,72^. Moreover, a TI vaccine could also be used in vaccination against self-antigens such as amyloid-β, where it is important to avoid elicitation of cytotoxic T cells that may cause unwanted immune pathology^73^. TI vaccines may also prove useful in diseases where rapid immune protection is needed, but where conventional TD vaccines are not recommended, justified, or efficient, e.g., for the protection against pathogens during epidemic infectious outbreaks or against bioterrorism^19,74,75^. Vaccine strategies without T-cell involvement have not yet been explored, perhaps because TI peptide antigens have been believed to lack immunoglobulin class switch and memory B cells^21^. In light of the growing body of evidence on protective B-cell memory forming without CD4 T_H_ cells, the true potential of TI vaccines should be revisited. Here, the peptide- and MPLA-loaded liposomes may serve as a useful tool for investigating further the characteristics of TI memory and the potential of such immune responses in disease and vaccination.

## Methods

### Mice

BALB/c (BALB/cOlaHsd, H-2^d^), C57BL/6 (C57BL/6JOlaHsd, H-2^b^) and athymic nude (Hsd:AthymicNude-Fox1) mice were purchased from Envigo (Horst, the Netherlands). CBA (CBA/J, Pde6b^rd1^), BTK (CBA/CaHN-Btk^xid^/J) and caspase-1 deficient (6 N.129S2-Casp1tm1Flv/J) mice were purchased from Jackson Laboratories (Bar Harbor, Maine, USA). TLR4- (B6-TLR4^tm1Aki^), MHC class II- (B6-I-Aa^tm1Blt^) and TCR-deficient (B6.129P2-Tcrb^tm1Mom^ Tcrd^tm1Mom^) mice were obtained from the Swiss immunological mouse repository (SWIMMR). Mice deficient for the inflammasome adopter protein ASC were originally from Genentech (San Francisco, CA)^76^. Mice were kept and bred under SPF conditions, in individually ventilated cages, in groups of 3–5 mice, at 21 °C, and with a 12 h–12 h light dark cycle at the animal facility of the *Biologisches Zentrallabor*, University Hospital Zurich. Primarily, female mice were used in the experiments that took place at daytime and under laminar. When testing mice at age below three weeks, both sexes were used. Mice were euthanized at endpoint using the carbon dioxide method. All procedures were approved by the animal Research Ethics Board and the Zurich cantonal veterinary office (licenses ZH 98/2014 and ZH 128/2017), and the ARRIVE guidelines for animal experiments were followed. No anesthesia or analgesia were applied. Mice were euthanized at endpoint using carbon dioxide. The primary endpoint used were measurements of antibodies to vaccine peptides. Other experimental endpoints were measurement of cytokines, protein expression on cells, mRNA expression, body temperature upon allergen challenge, and bacterial growth upon infection (cf. below).

### Preparation of peptide liposomes

Liposomes were prepared from dimyristoyl phosphatidyl choline (DMPC), dimyristoyl phosphatidyl glycerol (DMPG), cholesterol (Solvay, Brussels, Belgium), and monophosphoryl lipid A (MPLA; Avanti Polar Lipids, Alabaster, USA), as previously described^77^, and as illustrated in Fig. 1a. Chicken ovalbumin (OVA) was used as antigen, whereby short peptides thereof were designed for formulation in the liposomes. The peptide design took into consideration immunological and physicochemical characteristics (Table 1), thereby avoiding known T-cell binding and IgE-binding epitopes, but including IgG-binding epitopes^78–84^. The chosen OVA-derived sequences were synthesized and flanked with two palmitoylated lysines (Lys) on each terminal side of the peptide (PolyPeptide Laboratories, Strasbourg, France). The liposomes were loaded with 101–648 µg/ml peptide and 56–169 µg/ml MPLA. A liposomal preparation containing MPLA and peptide is hereafter named Lip-OVA_x-y_, *x-y* being the amino acid sequence of the OVA peptide^34^.

**Table 1:** Peptides from ovalbumin (OVA) formulated in liposomes

Code	Amino acids	Sequence OVA aa	Length (aa)	Aliphatic Index^a^	Instability Index^b^
OVA_15-29_	H-Lys(Pal)-Lys(Pal)-**FKELKVHHANENIFY**-Lys(Pal)-Lys(Pal)-NH2	15–29	15 + 4	61.58	32.91
OVA_58-72_	H- Lys (Pal)- Lys (Pal)-**RFDKLPGFGDSIEAQ**-Lys(Pal)-Lys(Pal)-NH2	58–72	15 + 4	46.32	67.59
OVA_91-150_	H-Lys(Pal)-Lys(Pal)-**TKPNDVYSFSLASRY**^c^-Lys(Pal)-Lys(Pal)-NH2	91–105	15 + 4	41.05	7.71
OVA_158-171_	H-Lys(Pal)-Lys(Pal)-**RNVLQPSSVDSQTA**-Lys(Pal)-Lys(Pal)-NH2	158–171	14 + 4	59.44	97.67
OVA_158-175_	H-Lys(Pal)-Lys(Pal)-**RNVLQPSSVDSQTAMVLV**-Lys(Pal)-Lys(Pal)-NH2	158–175	18 + 4	92.73	80.42
OVA_179-195_	H-Lys(Pal)-Lys(Pal)-**VFKGLWEKAFKDEDTQA**-Lys(Pal)-Lys(Pal)-NH2	179–195	17 + 4	41.9	-3.18

^a^The aliphatic index is regarded as a positive factor for solubility of proteins^92^.

^b^The instability index has been shown to inversely correlate with in vivo half-life^92^.

^c^Original sequence was TKPNDVYSFSLASRLY.

Characteristics of short OVA peptides synthesized and tested for T-cell independent immunization. Exclusion criteria for the peptide selection were known T-cell or IgE-binding epitopes while peptides with known IgG-binding epitopes were included. Minor amino acid mutations were created to assure peptide stability and solubility. The peptides were synthesized and flanked with two palmitoylated amino acids (Lys) on both the N- and C-terminal side. The fatty acid-derived palmitoyl group facilitated anchoring of the peptide in the bilayer of liposomes. The N-terminal amino group of all the peptides was kept free while the C-terminal carboxylic group was capped as amide.

### Immunization of mice

Mice were immunized subcutaneously (s.c.) in the scruff of the neck using syringes with 28 or 31 G needles. A single dose of the liposomal preparations ranged from 1 to 36 µg OVA peptide and 0.65–25.6 µg MPLA, depending on the age of the mice. Mice that were younger than three weeks were immunized while being with mothers, and the pups were subject to only a single injection as to minimize any health or stress burden to pups and mothers. The liposomes were diluted in phosphate-buffered saline (PBS) prior to the injection. TD control vaccines were made using full OVA protein (Grade V) from Sigma-Aldrich (Buchs, Switzerland) mixed with aluminum hydroxide (alum; Alhydrogel 2%) from Brenntag (Ballerup, Denmark). OVA in PBS was allowed to absorb on alum for one hour at room temperature. The OVA doses ranged from 10 to 100 µg OVA and the alum dose was kept at 1 mg. When indicated, the immunization was repeated.

### Measurement of OVA-specific antibody responses by ELISA

Mice were bled from the tail vein and whole OVA protein-specific IgM, IgG1, IgG2a, IgG2b, IgG3, and IgE antibody responses were determined by a sandwich ELISA^85^. Briefly, the plates were coated with 50 µl of 4 µg/ml OVA for IgM and IgG subclass detection and with anti-mouse IgE (#MCA 419, dilution 3:1000) from Bio-Rad for detection of IgE. OVA-specific IgE was detected with an in-house biotin-conjugated OVA (1:1000), while IgM and IgG subclasses were detected with biotin-conjugated goat anti-mouse IgM (#553403, 1:500), IgG1 (#553500, 1:1000) or IgG2a (#553502, 1:000) from BD Pharmingen or IgG2b (#AB97248, 1:1000) or IgG3 (#AB97258, 1:1000) from Abcam. All plates were developed with streptavidin-conjugated horseradish peroxidase (#405210, 1:000, Biolegen) and with TMB substrate (Invitrogen). Reactions were stopped with 25 µL of 2 N sulfuric acid (H_2_SO_4_) and absorbance as optical density (OD) read at 450 nm on a plate reader (BioTek Instruments, Winooski, VT). Antibody titers were defined as the highest dilution with absorbance two times that of sera from untreated mice. Alternatively, the OD at a given, non-saturated serum dilution was measured for IgG2b, IgG3 and IgM, while the serum concentration of OVA-specific antibodies was calculated against standards for IgG1, IgG2a (BioLegend) and IgE (AbD Serotec)^34^.

### ELISA for avidity measurements

The avidity of serum antibodies were measured in three steps as previously described^86,87^. Firstly, the above-described ELISA was optimized so that the amount of serum antibody binding the OVA-coated plate represented a small portion of the amount of antibody in the non-binding liquid phase. The amount was equivalent to a developed ELISA of OD 0.2. Secondly, the test sera was diluted to obtain the optimal antibody concentration (OD 0.2). Thirdly, the diluted sera were incubated at room temperature with OVA protein at a range of OVA concentrations and for 2 h. The sera were then transferred to OVA-coated plates and the ELISA developed. The concentration of OVA needed to inhibit 50% of the antibodies from binding to the plate (IC_50_) was calculated by “log (inhibitor) vs response” fitting using GraphPad Prism 7.02 (GraphPad Software Inc., La Jolla, CA). Sera from individual mice within each treatment group was pooled to yield one sample per group.

### Germinal center analysis by immunohistochemistry, immunofluorescence and flow cytometry

BALB/c or athymic nude mice were immunized with 10 µg peptide-loaded liposomes. As a positive control for GC formation, 10 µg OVA on alum was administered to BALB/c mice^88^. Spleens were harvested for the analysis of GCs on various time intervals after injection, and the tissues were snap-frozen for immunohistochemistry (IHC) or immunofluorescence (IF). The frozen sections were stained with peanut agglutinin (PNA) and anti-B220 (#553084, BD Bioscience) or with PNA-Dylight549 (#BA-0074, Vector Labs), anti-B220-Dylight488 (#18120340, FisherScientific) and DAPI. All staining was outsourced to Sophistolab AG (Muttenz, Switzerland).

Alternatively, spleens were harvested seven days after injection with peptide-loaded liposomes and analyzed by flow cytometry after RBC lysis, FcR blocking with anti-CD16/32 antibodies, and staining with fluorescently labeled anti-B220 (#11-0452-82, dilution 1:300), anti-GL-7 (#12-5902-82, 1:200), and anti-CD38 (#17-0381-82, 1:200) from eBioscience. No antigen-specific antibody was applied. Germinal center B cells were defined as positive for B220 and GL-7 and negative for CD38. As a TD control vaccine, OVA-Alu was used. Acquisition was done with FACSCanto (BD Bioscience, Basel, Switzerland) and analysis with FlowJo 10.0.8 (FlowJo, LLC, Ashland, OR).

### In vitro stimulation of B cells

Spleens and lymph nodes were harvested from wild type or genetically modified mice. Single cells suspension prepared and red blood cells lysed. B cells were purified using CD19 MicroBeads (#130-121-301 from Miltenyi Biotec), the amount of CD19^+^ cells typically being >90%. The cells in supplemented medium were stimulated with 10 µg/ml Lip-OVA_58-72_ for two days. Cells were harvested to measure the IgM expression by PCR after mRNA islation using RevertAid First Strand cDNA Synthesis Kit (Thermo Fisher Scientific). One microgram RNA was reverse transcribed to cDNA using RT2 first-strand kit (Thermo Fisher Scientific). Real-time PCR was performed with Light Cycler 480 and SYBR Green Master (Roche) using the IgM 5’-3’primers from Microsynth (Balgach, Switzerland): fwd. GACAAGTCCACTGGTAAACCC, rev. CCGCCTGTGTCAGACATGA. Cell supernatants were analyzed for total IgM and for TNF-α and IL-10 secretion by ELISA according to the provider (Invitrogen).

In part, cells were cultured with the immune suppressive agents dexamethasone (Mephameson from Mepha Pharma), tacrolimus (Prograf Inf from Astellas Pharma) or cyclosporine A (Sandimmun Inf from Novartis) or the pan-caspases inhibitor Z-VAD-fmk (Selleck Chemicals) in combination with Lip-OVA_58-72_ for B cells or concanavaline A (Cayman chemicals) for spleen cells.

### Induction and monitoring of systemic allergic anaphylaxis

BALB/c mice were immunized s.c. with 10 µg Lip-OVA_58-72_ or with 100 µg OVA-Alu as described above. A booster was given four weeks later. On weeks 8 and 11, all mice received s.c. injections of 100 µg OVA on alum in 100 µL PBS as to produce OVA-specific IgE sensitization. On week 14, mice were challenged by intraperitoneal (i.p.) injection of 50 µg OVA in 100 µl PBS, and anaphylaxis was analyzed by measuring the rectal temperature before and after the challenge using a digital thermometer (Thermalert TH-5 with a RET-3 probe, Physitemp, Huron, NJ) as described^89^. Alternatively, athymic nude mice were immunized with 10 µg Lip-OVA_58-72_ twice with 4-week intervals. Three weeks later, the mice were adoptively transferred with serum from IgE-sensitized BALB/c mice. Next day, the nude mice were challenged for induction of anaphylaxis. A control group of athymic nude mice was not immunized prior to serum transfer.

### Infection of mice with *Listeria monocytogenes*

BALB/c mice were immunized s.c. with 10 µg Lip-OVA_58-72_. One to three weeks after the last immunization, the mice were infected with OVA-expressing and streptomycin-resistant *Listeria monocytogenes*^90^. After growing bacterial stocks to mid log phase in brain-heart infusion (BHI) broth, mice received 10,000 colony-forming units (CFU) in PBS by intravenous injection, and the bacterial burden in spleen and in one liver lobe was assessed after 48 h by growing serial dilutions of saponin-treated organ homogenates on BHI agarose plates supplemented with 200 µg/mL streptomycin^91^. After 48 h of incubation at 37 °C, the CFU were counted and the bacterial burden calculated.

### Statistical analysis

The statistical analysis were performed using GraphPad Prism. The non-parametric data were analyzed by Kruskal-Wallis analysis for multiple comparisons or by two-tailed Mann-Whitney analysis for analysis of two groups. Corrections for multiple comparisons were performed as suggested by GraphPad Prism, e.g., Dunn’s correction with Kruskal-Wallis. For antibody responses over time, the area under the curve for each group was calculated and compared statistically. Some experiments were analyzed using a Mixed-effect model with Geisser-Greenhouse corrections. Typically, means and standard deviations (SD) or the means are shown. For logarithmic data (antibody titers), geometric means and geometric standards deviations (GSD) are shown. All experiments were repeated at least once.

### Reporting summary

Further information on research design is available in the Nature Research Reporting Summary linked to this article.

### Supplementary information

Supplementary Information

Reporting Summary

## Data Availability

Data supporting the findings of this study are available in this paper, Supplementary information, or are available from the corresponding author upon request.
