# Supplementary material for: T cell independent antibody responses with class switch and memory using peptides anchored on liposomes
Source: NPJ Vaccines. 2024 Jun 22;9:115. doi: 10.1038/s41541-024-00902-3 (PMC11193769; doi:10.1038/s41541-024-00902-3)
Supplement: Supplementary file 1 — Supplementary Information [file 41541_2024_902_MOESM1_ESM.pdf]

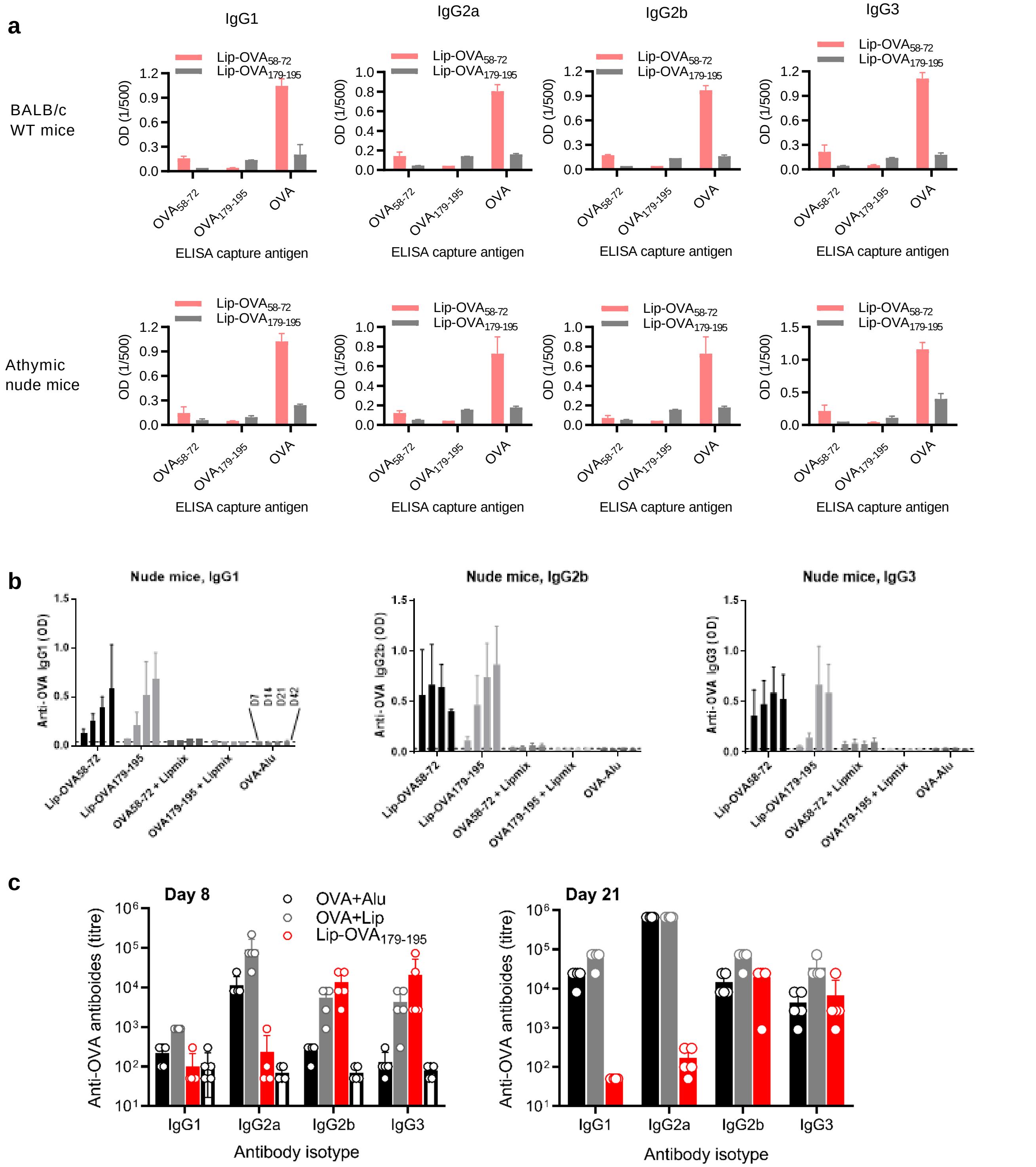

Supplementary Figure 1. OVA-specific antibody responses in WT BALB/c and athymic nude mice after immunization with OVA-derived peptides and MPLA in loaded on the liposomes.
(a) BALB/c or athymic nude mice (n=4-6 per group) were subcutaneously immunized with 10 µg Lip-OVA$_{58-72}$ or Lip-OVA$_{179-195}$ or with OVA-Alu on days 0 and 7. Six weeks after immunization, antibodies in mouse sera (1:500 dilution) specific for the peptides (OVA$_{58-72}$ or OVA$_{179-195}$) or the full protein (OVA) were measured with ELISA. (**b**) Athymic nude mice (n=3) were subcutaneously immunized with 10 µg Lip-OVA$_{58-72}$ or Lip-OVA$_{179-195}$ or with 10 µg OVA$_{58-72}$ or OVA$_{179-195}$ dissolved in PBS and mixed with MPLA-containing liposomes on days 0 and 7. Protein OVA with Alum was used as TD control. On days 7, 14, 21 and 42 blood was collected and analyzed for OVA-specific antibodies by ELISA (1:250 dilution). (**c**) BALB/c mice (n=5) were immunized with 10 µg Lip-OVA$_{179-195}$ or the same amount OVA protein mixed with MPLA-containing liposomes (OVA+ Lip) or OVA mixed with alum (OVA+Alu). Antibodies in serum was analyzed 5 and 21 days after immunization.

T-cell independent liposomal peptide vaccines
Hjalmsdottir et al.

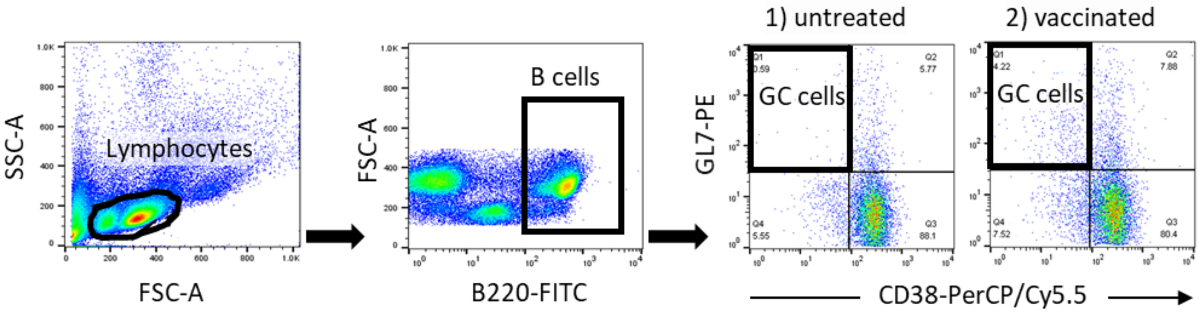

Supplementary Figure 2. Gating strategy for analysis of germinal center (GC) cells by flow cytometry as described in Figure 2d.
Mice were vaccinated or not with Lip-$OVA_{58\text{-}72}$. Blood or spleen was collected and single cell suspension prepared for flow cytometry. The lymphocytes were identified by forward scatter and side scatter properties. B cells were identified using anti-B220 (CD45R) antibodies. Germinal center cells were identified based on expression of CD38 (negative in GC cells) and GL7 (positive in GC cells).

T-cell independent liposomal peptide vaccines
Hjalmsdottir et al.

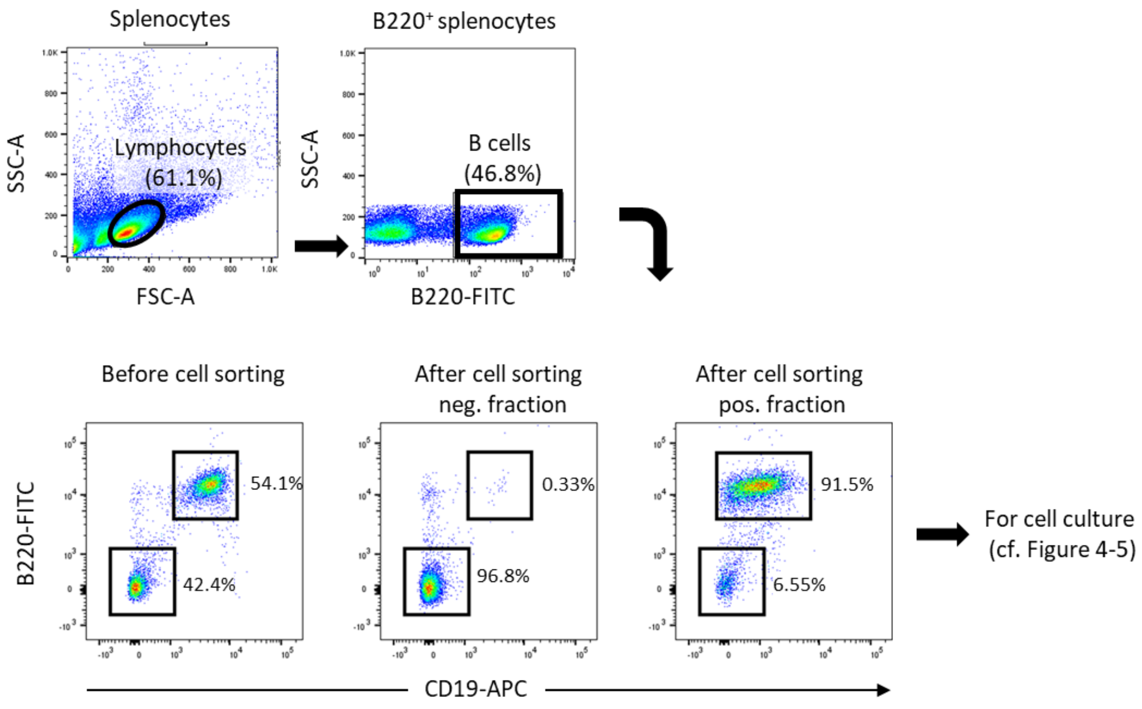

Supplementary Figure 3. Sorting analysis of splenocytes used for in vitro analysis described in Figure 4 an Figure 5.
Spleen were was collected from naïve mice and single cell suspension prepared for flow cytometry. The lymphocytes were identified by forward scatter and side scatter properties. B cells were identified using anti-B220 (CD45R) antibodies. The frequency of B220 B cells was analyzed prior to magnetic sorting with anti-CD19 beads as well as after the magnetic sorting. For the latter, both the CD19-negative fraction (flow through) and the CD19-positive fraction were measured.

T-cell independent liposomal peptide vaccines
Hjalmsdottir et al.

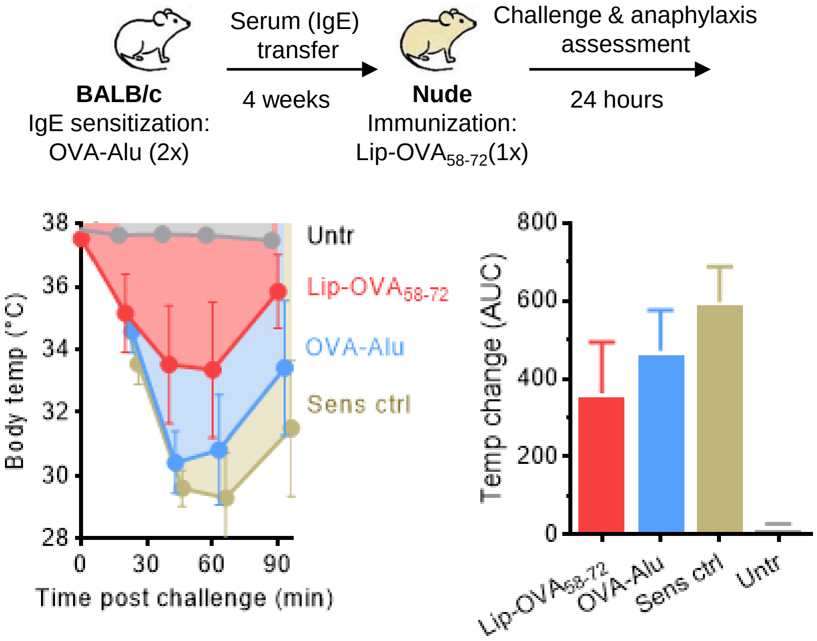

Supplementary Figure 4. Prophylactic immunization with Lip-OVA$_{58-72}$ reduced anaphylactic responses in OVA-allergic mice.
Nude mice (n=5) were injected twice with Lip-OVA$_{58-72}$ or OVA-Alu. Three weeks later, the nude mice received a transfer of serum from IgE-sensitized BALB/c mice. Next day, the nude mice were challenged with OVA. The body temperature upon challenge was monitored as a measure for allergic anaphylaxis, and the AUC for the body-temperature curves was calculated using pre-challenge temperature as baseline. Nude mice that were no immunized with Lip-OVA$_{58-72}$ served as controls. All results are expressed as mean + SEM.

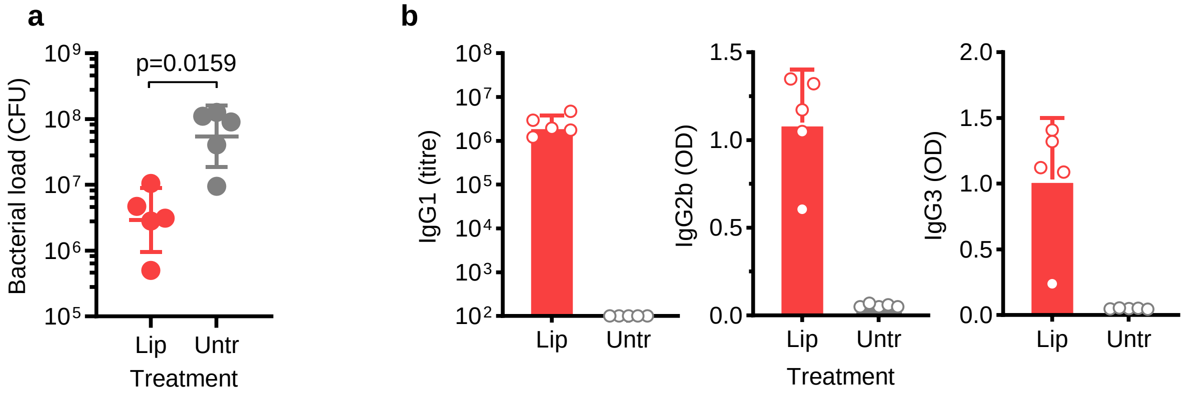

Supplementary Figure 5. Immunization with liposomal TI vaccines controls *Listeria* bacterium infection in mice.
BALB/c mice (n=5) were immunized with a combination of Lip-OVA$_{58-72}$ and Lip-OVA$_{179-195}$. Six months later, the mice were infected with OVA-expressing *Listeria monocytogenes*. (**a**) The bacterial burden in liver was analyzed 48 hours after infection and compared with infected but non-immunized mice. Results were analyzed by Mann Whitney U tests. (**b**) On the day of infection, mice were also bled for analysis of OVA-specific antibodies. Geometric means are shown for CFU data and IgG1 titers. Means and SEM are shown for IgG2b and IgG3 data.
